# Patient-reported outcomes and target effect sizes in pragmatic randomized trials in ClinicalTrials.gov: A cross-sectional analysis

**DOI:** 10.1371/journal.pmed.1003896

**Published:** 2022-02-08

**Authors:** Shelley Vanderhout, Dean A. Fergusson, Jonathan A. Cook, Monica Taljaard

**Affiliations:** 1 Clinical Epidemiology Program, Ottawa Hospital Research Institute, Ottawa, Ontario, Canada; 2 School of Epidemiology and Public Health, University of Ottawa, Ottawa, Ontario, Canada; 3 Department of Medicine, University of Ottawa, Ottawa, Ontario, Canada; 4 Centre for Statistics in Medicine & Oxford Clinical Trials Research Unit, Nuffield Department of Orthopaedics, Rheumatology and Musculoskeletal Sciences, University of Oxford, Oxford, United Kingdom; Washington University in St Louis School of Medicine, UNITED STATES

## Abstract

**Background:**

Use of patient-reported outcomes (PROs) and patient and public engagement are critical ingredients of pragmatic trials, which are intended to be patient centered. Engagement of patients and members of the public in selecting the primary trial outcome and determining the target difference can better ensure that the trial is designed to inform the decisions of those who ultimately stand to benefit. However, to the best of our knowledge, the use and reporting of PROs and patient and public engagement in pragmatic trials have not been described. The objectives of this study were to review a sample of pragmatic trials to describe (1) the prevalence of reporting patient and public engagement; (2) the prevalence and types of PROs used; (3) how its use varies across trial characteristics; and (4) how sample sizes and target differences are determined for trials with primary PROs.

**Methods and findings:**

This was a methodological review of primary reports of pragmatic trials. We used a published electronic search filter in MEDLINE to identify pragmatic trials, published in English between January 1, 2014 and April 3, 2019; we identified the subset that were registered in ClinicalTrials.gov and explicitly labeled as pragmatic. Trial descriptors were downloaded from ClinicalTrials.gov; information about PROs and sample size calculations were extracted from the manuscript. Chi-squared, Cochran–Armitage, and Wilcoxon rank sum tests were used to examine associations between trial characteristics and use of PROs. Among 4,337 identified primary trial reports, 1,988 were registered in CT.gov, of which 415 were explicitly labeled as pragmatic. Use of patient and public engagement was identified in 39 (9.4%). PROs were measured in 235 (56.6%): 144 (34.7%) used PROs as primary outcomes and 91 (21.9%) as only secondary outcomes. Primary PROs were symptoms (64; 44%), health behaviors (36; 25.0%), quality of life (17; 11.8%), functional status (16; 11.1%), and patient experience (10; 6.9%). Trial characteristics with lower prevalence of use of PROs included being conducted exclusively in children or adults over age 65 years, cluster randomization, recruitment in low- and middle-income countries, and primary purpose of prevention; trials conducted in Europe had the highest prevalence of PROs. For the 144 trials with a primary PRO, 117 (81.3%) reported a sample size calculation for that outcome; of these, 71 (60.7%) justified the choice of target difference, most commonly, using estimates from pilot studies (31; 26.5%), standardized effect sizes (20; 17.1%), or evidence reviews (16; 13.7%); patient or stakeholder opinions were used to justify the target difference in 8 (6.8%). Limitations of this study are the need for trials to be registered in ClinicalTrials.gov, which may have reduced generalizability, and extracting information only from the primary trial report.

**Conclusions:**

In this study, we observed that pragmatic trials rarely report patient and public engagement and do not commonly use PROs as primary outcomes. When provided, target differences are often not justified and rarely informed by patients and stakeholders. Research funders, scientific journals, and institutions should support trialists to incorporate patient engagement to fulfill the mandate of pragmatic trials to be patient centered.

## Abstract 2

In a cross-sectional analysis, Shelley Vanderhout and colleagues assess the reporting of patient reported outcomes and target effect sizes in pragmatic randomized trials in ClinicalTrials.gov.

## Author summary

**Why was this study done?:**

- Pragmatic trials aim to answer real-world questions that are meaningful to patients and can be applied to healthcare settings.
- Measuring outcomes that are important from the patient perspective (such as quality of life, patient experience of healthcare, or pain; referred to as patient reported outcomes) and including patients as partners in research (referred to as patient engagement) can help studies to be more patient centered.
- How often and in what circumstances researchers use patient reported outcomes or patient engagement in pragmatic trials have not been described.

**What did the researchers do and find?:**

- We searched published reports about trials that were likely to be pragmatic, published in English between 2014 and 2019, and registered in a major clinical trials registry called ClinicalTrials.gov.
- Out of 415 trials identified, few used patient engagement and main trial outcomes were often not patient-reported outcomes (PROs).
- Limitations of this study are exclusion of trial reports in languages other than English, and those not registered at ClinicalTrials.gov, which may have lowered the completeness of our analysis.

**What do these findings mean?:**

- Pragmatic trials are meant to reflect patient values and priorities, but our research demonstrates that there is room for improvement to accomplish this.
- Research funding agencies, research institutions, and scientific journals should encourage researchers to include PROs in pragmatic trials going forward to keep the focus of research on patient values.

## Introduction

Pragmatic randomized controlled trials (RCTs) aim to inform decisions and generate evidence directly applicable to clinical practice by adopting study settings and methods, which are similar to usual care, and evaluating interventions using criteria that account for the interests of patients [1]. Measuring patient-important outcomes is a key characteristic of pragmatic trials [2], and patient and public engagement in research can improve its relevance to end users [3]. Patient-reported outcomes (PROs) are considered to be important to patients as they are unique indicators of disease experience, patient empowerment, treatment efficacy, and healthcare quality [4]. The International Society for Quality of Life Research (ISOQOL) defines PROs as “measurement[s] of any aspect of a patient’s health that come directly from the patient, without interpretation of the patient’s response by a physician or anyone else” [5]. According to the Patient-Reported Outcome Measures Information System (PROMIS), PROs can be categorized as quality of life or health-related quality of life (QOL or HRQOL), functional status, symptoms or symptom burden, health behaviors, and patient experience [6]. PROs are patient centered, hold potential to guide clinical decision-making, and are therefore well suited to pragmatic trials.

The SPIRIT group has provided SPIRIT-PRO guidelines for protocols of RCTs measuring PROs, which include specifying the rationale for the choice of PRO, use of patient and public engagement, and whether the PRO was used to determine the target sample size [7]. Similar guidance for reporting on RCTs measuring PROs is available from CONSORT [8]. The PROTEUS Consortium [9] has also curated resources for designing, analyzing, reporting, and interpreting studies with PROs. Considering patient perspectives to ensure that PROs are indeed relevant and selected appropriately can impact the potential for trial evidence to guide clinical decision-making. Clear communication about how the target sample size was determined and informed by the PRO and whether or how patients were engaged in determining the target difference affects the interpretation and applicability of study results [10]. However, as far as we are aware, there is no formal guidance for including patients in establishing the target difference in sample size calculations [11]. Previous reviews have explored use of PROs in trials within specific clinical areas such as cancer [12,13] and joint health [14], and searches of trials registered in ClinicalTrials.gov have been conducted to describe use of PROs between 2004 and 2007 [15] and 2007 and 2013 [16]. To the best of our knowledge, the extent to which PROs are included in pragmatic trials and how this varies across study characteristics, and the extent to which patients are engaged in study design considerations such as the choice of target difference used in sample size calculations, have not previously been described.

The objectives of this study were to review a sample of pragmatic trials to determine the following:

1. The prevalence of reporting on patient, public, and other stakeholder engagement;
2. The prevalence and types, according to PROMIS [6] categories, of PROs used as primary or coprimary, and secondary outcomes;
3. Whether the use of PROs as (a) primary or coprimary outcome or (b) any type of outcome (whether primary or secondary) varies across trial characteristics such as patient age group, study setting, trial design, country, type and purpose of intervention, study funder, journal impact factor, use of patient or stakeholder engagement, and publication year;
4. How sample sizes and target differences are determined when PROs are primary or coprimary outcomes.

## Methods

This study is reported as per the extension to the PRISMA Statement for Reporting Literature Searches in Systematic Reviews (PRISMA-S) guideline (see S1 PRISMA Checklist).

### Study selection

This methodological review was part of a broader project to inform design and ethical considerations for pragmatic trials [17]. For this broader project, we developed and validated an electronic search filter [18], which was used in Ovid MEDLINE to identify RCTs more likely to be pragmatic and published in English between January 1, 2014 and April 3, 2019. The filter captures elements of reports indicative of more pragmatic trials including descriptors of trial design, setting, data collection, interventions and comparators, and outcomes. The search filter as well as inclusion and exclusion criteria for the larger study are summarized in S1 Search Filter and S1 Screening. Briefly: reports were eligible if they were the primary report of an RCT of a health or healthcare intervention with a target sample size of at least 100 individuals. Full details of the screening process and a descriptive analysis of the landscape of trials included in the database have been published elsewhere [19]. For feasibility reasons, we used the existing database rather than implement a new search: Due to its methodological nature, the results from this review would likely still be reflective of current practice. Furthermore, given the large number of retrieved trials and breadth of clinical areas, patient populations, and geographic regions, results from this review could serve as a useful baseline to which results from future reviews can be compared. For the present analysis, a subset of trials registered in ClinicalTrials.gov (CT.gov) and explicitly described as “pragmatic” anywhere in the full text was identified. We focused on trials registered in CT.gov to capitalize on the availability of descriptive information including trial purpose and study interventions. This methodological review was planned after creation of the larger database; the study protocol was specified in advance of the data extraction and is available in S1 Protocol. There were no deviations from the prespecified analysis plan.

### Downloaded data elements

Data for analysis were retrieved from ClinicalTrials.gov, Web of Science [20], Ovid MEDLINE, Journal Citation Reports (JCR), and full-text manuscripts. Clinicaltrials.gov is a registry of RCTs run by the US National Library of Medicine and includes a wide array of detailed trial information [21]. Precategorized descriptors obtained from ClinicalTrials.gov were participant age group (pediatric (≤18 years), >65 years, other, or mixture), primary purpose, and type of intervention. Primary purpose categories were treatment, prevention, diagnostic, supportive care, screening, health services research, or other. Intervention categories were drug, device, biological/vaccine, procedure/surgery, radiation, behavioral, genetic, dietary supplement, educational/behavioral, or other. Year of publication was downloaded from MEDLINE and journal impact factor from JCR (as of 2018). For a small number of studies with impact factors not available from JCR, values were imputed from the SCImago Journal and Country Rank (SJR) and Google searches.

### Manually extracted data

An extraction form (available in S1 Data Extraction Form) was created to obtain information from full-text review of each included trial report. Some elements included in this form were previously recorded by 7 members of the study team as part of the broader study. These elements were unit of randomization (individual or cluster), country of trial recruitment (Canada, USA, UK, other European country, Australia, low- or middle-income country (LMIC), other, or unclear), type of setting (primary care, hospital, nursing home, community, schools, workplace, or other), reporting of any patient or other stakeholder engagement, and use of PROs as primary, coprimary, or secondary outcomes. PROs were defined according to the Food and Drug Administration’s definition: “Any report of the subjective status of a patient’s health condition or response to an intervention that comes directly from the patient or their proxy, without interpretation of the patient’s response by a clinician or anyone else, for example, health-related quality of life, symptoms, severity, utilities, pain, or satisfaction” [22]. Patient or stakeholder engagement was identified through descriptions in manuscripts, author lists, and acknowledgments sections. Data were extracted from an initial subset of 25 trials by all 7 reviewers to ensure consistency, followed by individual reviewer extractions for the remainder of the trials.

The remaining data elements were extracted by one reviewer (SV) for all included trials. A second reviewer (MT) duplicated extractions for PRO and sample size information from a subset of 10 studies for training purposes. These 2 reviewers met regularly to discuss any potential problems or uncertainties in the extractions. Each trial was classified based on whether the authors clearly identified one or more primary trial outcomes, types of PROs used, if any, and whether any justifications were provided for their use. Types of PROs were classified according to PROMIS [6] categories (QOL or HRQOL, symptoms or symptom burden, functional status, health behaviors, patient experience, or other). Any provided justifications for use of PROs were identified in the descriptions of study outcomes and classified as using literature reference or explanation, patient consultation, other, or none. We also classified whether a sample size or power calculation was provided and, if so, for which outcome. Any reported adjustment for attrition was noted. For each trial with a sample size or power calculation, we classified what method was used for justifying the target difference using the categories in Cook and colleagues [23], namely anchor, distribution, health economic, patient or stakeholder consultation (“opinion seeking”), review of the evidence base, pilot or one prior study, standardized effect size, or other. The type of target difference was further classified as important, realistic, both important and realistic, or not specified or unclear. For example, trials that used evidence review or a pilot study to justify the target difference were classified as “realistic,” and those using patient/stakeholder opinion as “important.” Trials that simply calculated power based on an available sample size were classified as not providing any justification for the target difference. Finally, type of funder, if available, was classified as government, university, or international agency; foundation or special interest group; industry; or individual (multiple selections were possible). Statements about sample size or power were considered present if they were part of the manuscript text or supplements; references to study protocols or other publications were not reviewed.

All downloaded and manually extracted data were collated in Airtable, an online collaborative database that can be applied to a spreadsheet [24].

### Analysis

Trial characteristics were summarized descriptively using counts and percentages for categorical variables and median and interquartile range (Q1-Q3) for continuous variables. The prevalence and types of PROs used were tabulated using frequency distributions. To test associations between use of PROs and categorical study characteristics, chi-squared tests of independence were used. The primary analyses compared studies reporting PROs as primary or coprimary outcomes with those reporting PROs as secondary outcomes or not used. In secondary analyses, we grouped studies reporting PROs as either primary or secondary outcomes and compared with those not using PROs as outcomes. The strength of the association with each categorical study characteristic was described using prevalence (“risk”) ratios and 95% Wald confidence intervals. Study setting was dichotomized as clinical (primary care, hospital, or nursing home) versus nonclinical (schools, worksites, communities, or other). Country of study recruitment was dichotomized as LMIC versus non-LMIC or mixture. Study regions were classified as North America only, Europe only, or other or mixture. Intervention types were categorized as clinical (drug, device, biological, procedure, radiation, or genetic) versus dietary supplements or educational/behavioral versus other. Primary purpose was classified as treatment, prevention, health services research, or other. Because each trial could have multiple funding sources, we dichotomized each funding source as present or absent for analysis. Year of publication was collapsed into 2-year intervals and analyzed using a Cochran–Armitage (“chi-squared”) test for trend to determine if prevalence of PROs increased over time. For journal impact factor, a Wilcoxon rank sum test was used to compare the distribution of impact factors between the groups. All statistical tests were conducted in R version 4.0.4 [25] using a two-sided alpha level of 0.05.

### Patient and public involvement

There were no patients or members of the public involved in this analysis.

### Research ethics approval

This study did not include human research participants; therefore, research ethics approval was not required.

## Results

Of the existing database of 4,337 primary RCT reports, 1,988 were registered in Clinicaltrials.gov; of these, 415 explicitly referred to the design as pragmatic anywhere in the text and were included in this review.

### Trial characteristics

Trial descriptors are shown in Table 1. Half (207; 49.8%) of included trials were conducted in North America, a third (141; 34.0%) in Europe, and 10.8% (45) in LMICs. Study settings were primary care (127; 30.6%); hospital (192; 46.3%); public health including community, schools, or workplaces (51; 12.4%); or other (37; 8.9%). Most studies (263; 63.4%) were individually randomized, while 152 (36.6%) were cluster randomized. Common purposes of trial interventions were treatment (159; 38.3%), health services research (96; 23.1%), or prevention (81; 19.5%); most common interventions were educational or behavioral (171; 41.2%), drugs (53; 12.8%), procedure/surgery (38; 9.2%), or device (35; 8.4%). Study funding most often came from governments, universities, or international agencies (321; 77.3%) followed by foundations or special interest groups (106; 25.5%) and industry (75; 18.1%). The median journal impact factor was 5.4 (Q1-Q3: 3.5 to 19.0).

**Table 1:** General characteristics of trials included in the review and reporting of stakeholder engagement (*N =* 415).

Characteristic	Frequency (%)^a^
**Country of trial recruitment**^b^ Canada United States of America United Kingdom European Union Australia or New Zealand LMIC Other high-income country	45 (10.8%) 162 (39.0%) 22 (5.3%) 119 (28.7%) 6 (1.4%) 45 (10.8%) 49 (11.8%)
**Type of setting** Clinical: primary care Clinical: hospital care Clinical: nursing homes Public health: community or residential setting Public health: schools Public health: workplaces Mixture, unclear, or other	127 (30.6%) 192 (46.3%) 8 (1.9%) 43 (10.4%) 4 (1.0%) 4 (1.0%) 37 (8.9%)
**Trial design (unit of randomization)** Individually randomized Cluster randomized	263 (63.4%) 152 (36.6%)
**Primary purpose (based on** **ClinicalTrials.gov****)**^c^ Treatment Prevention Diagnostic Supportive care Screening Health services research Other	159 (38.3%) 81 (19.5%) 21 (5.1%) 39 (9.4%) 14 (3.4%) 96 (23.1%) 5 (1.2%)
**Types of experimental interventions**^b^^,^^c^^,^^d^ Drug Device Biological/vaccine Procedure/surgery Radiation Genetic Dietary supplement Educational or behavioral Other	53 (12.8%) 35 (8.4%) 3 (0.7%) 38 (9.2%) 1 (0.2%) 1 (0.2%) 1 (0.2%) 171 (41.2%) 142 (34.2%)
**Type of funder**^b^ Government, university, or international agency Foundation or special interest group Industry Unfunded Individual No funding information provided	321 (77.3%) 106 (25.5%) 75 (18.1%) 5 (1.2%) 2 (0.5%) 13 (3.1%)
**Journal impact factor**^e^ Min, max Median (Q1-Q3)	0.48, 70.7 5.4 (3.5–19.0)
**Reported patient or public engagement** Yes No Unclear	39 (9.4%) 370 (89.2%) 6 (1.4%)
**Reported any other stakeholder engagement** Yes No Unclear	51 (12.3%) 356 (85.8%) 8 (1.9%)

^a^Unless otherwise indicated.

^b^Multiple selections possible.

^c^Approximately 12 trials with missing values in ClinicalTrials.gov were manually classified by extractors; 3 trials with obviously erroneous ClinicalTrials.gov values were manually classified by extractors.

^d^One trial with missing ClinicalTrials.gov intervention was reclassified by extractors.

^e^Impact factors for 19 trials were not available and 16 were imputed using the SJR.

LMIC, low- or middle-income country; SJR, SCImago Journal and Country Rank.

### Reporting of patient and stakeholder engagement

Patient and public engagement (Table 1) was identified in 39 trials (9.4%), either through explicit statements within trial descriptions or through trial authorship or acknowledgements; other stakeholder engagement was identified in 51 (12.3%), including knowledge users, decision-makers, or policy makers.

### Use of PROs

Details about the use of PROs are presented in Table 2. Among the 415 trials, 235 (56.6%) measured PROs: 144 (34.7%) used PROs as primary or coprimary outcomes and 91 (21.9%) used PROs as only secondary outcomes. A small number of studies (56; 13.5%) had more than one coprimary outcome; of these, 10 studies used a mixture of PROs and clinical outcomes. Primary or coprimary outcome PROs were most frequently symptoms (64; 44.4%), followed by health behaviors (36; 25.0%), QOL or HRQOL (17; 11.8%), functional status (16; 11.1%), and patient experience (10; 6.9%). A rationale for the chosen PRO was provided in 24 (16.7%) trials; these were commonly based on literature or explanation (20; 13.8%), patient consultation (3; 2.1%), or other stakeholder consultation (1; 0.7%).

**Table 2:** Characteristics of PROs in pragmatic trials (*N =* 415).

Characteristic	Frequency (%)^a^
**Was the primary outcome clearly identified?** Yes (one primary) Yes (multiple coprimaries) No or unclear	330 (79.5%) 56 (13.5%) 29 (7.0%)
**Does the trial include PROs as primary or secondary outcomes?** Yes As a primary or coprimary outcome As a secondary outcome (not primary) No Unclear	235 (56.6%) 144 (34.7%) 91 (21.9%) 178 (42.9%) 2 (0.5%)
**If trial had coprimary outcomes (*N* = 56), were they a mix of PRO and clinical?** Yes No	10 (17.9%) 46 (82.1%)
**What type of PRO was used?**^a^ **Among trials using PROs as either primary or secondary outcome (*N =* 235)** QOL or HRQOL Symptoms or burden of symptoms Functional status Health behaviors Patient experience Other **Among trials with PRO as a primary or coprimary outcome (*N* = 144)** QOL or HRQOL Symptoms or burden of symptoms Functional status Health behaviors Patient experience Other **Among trials with PRO as only a secondary outcome (*N* = 91)** QOL or HRQOL Symptoms or burden of symptoms Functional status Health behaviors Patient experience Other	63 (26.8%) 102 (43.4%) 35 (15.3%) 65 (27.7%) 39 (16.6%) 38 (16.2%) 17 (11.8%) 64 (44.4%) 16 (11.1%) 36 (25.0%) 10 (6.9%) 18 (12.5%) 46 (50.5%) 38 (41.8%) 20 (22.0%) 29 (31.9%) 19 (20.9%) 20 (22.0%)
**Was a justification provided for the PRO when a primary or coprimary outcome? (*N* = 144)** Yes Literature reference or explanation Patient consultation Other stakeholder consultation No	24 (16.7%) 20 (13.8%) 3 (2.1%) 1 (0.7%) 122 (84.7%)

^a^Multiple selections were possible as a trial could have multiple secondary outcomes; all possible secondary outcomes were classified.

PRO, patient-reported outcome; QOL or HRQOL, quality of life or health-related quality of life.

### Variation in use of PROs across trial characteristics

The analysis comparing characteristics of trials using PROs as primary or coprimary outcomes (versus secondary outcomes or not used) is presented in Table 3. We found statistically significant differences based on trial age group, trial design, country of recruitment, geographic region, type of intervention, primary purpose, type of funder, and journal impact factor. Trial characteristics with lower prevalence of use of PROs were as follows: conducted exclusively in children or exclusively in adults over 65 years, use of cluster randomization, recruitment in LMIC settings, testing clinical interventions, primary purpose of prevention, receiving industry funding, and higher journal impact factor. Trials conducted exclusively in Europe had the highest prevalence of use of PROs. Prevalence of PROs was not significantly associated with other stakeholder engagement, and there did not seem to be a significant trend toward improvement over time. Table 4 shows the results of the analyses comparing studies that used PROs as any outcome to studies that did not use PROs; most conclusions remained the same, except type of intervention was no longer significantly associated with use of PROs. While the observed prevalence of using PROs was higher among trials reporting patient or public engagement (66.7% versus 55.8%), the confidence interval around the prevalence ratio was wide and the association was not statistically significant.

**Table 3:** Trial characteristics associated with use of PROs as primary or coprimary outcomes versus secondary outcomes only or not used.

	PROs as primary or coprimary outcome (*N =* 144)	PROs secondary or not used^a^ (*N* = 271)	Prevalence ratio (95% CI)	*P* value^b^
**Trial age group** ≤18 years >65 years Other or mixture	9 (27.3%) 8 (16.0%) 127 (38.3%)	24 (72.7%) 42 (84.0%) 205 (61.7%)	0.71 (0.40 to 1.27) 0.42 (0.22 to 0.80) Reference	0.0056
**Study setting** Clinical Nonclinical or other	114 (35.7%) 30 (31.3%)	205 (64.3%) 66 (68.8%)	1.14 (0.82 to 1.59) Reference	0.4181
**Trial design** Individually randomized Cluster randomized	108 (41.1%) 36 (23.7%)	155 (58.9%) 116 (76.3%)	1.73 (1.26 to 2.39) Reference	0.0003
**Country of recruitment** LMIC only Non-LMIC or mixture	5 (11.1%) 139 (37.6%)	40 (89.9%) 231 (62.4%)	0.30 (0.13 to 0.68) Reference	0.0004
**Region** North America only Europe only Other or mixture	69 (35.6%) 52 (41.3%) 23 (24.2%)	125 (64.4%) 74 (58.7%) 72 (75.8%)	1.47 (0.98 to 2.20) 1.70 (1.13 to 2,57) Reference	0.0291
**Type of intervention**^c^ Clinical Dietary or behavioral Other or mixture	27 (24.3%) 62 (40.0%) 55 (36.9%)	84 (75.7%) 93 (60.0%) 94 (63.1%)	0.66 (0.45 to 0.97) 1.08 (0.81 to 1.44) Reference	0.0233
**Primary purpose**^d^ Treatment Prevention Health services research Other	75 (47.2%) 12 (14.8%) 32 (33.3%) 25 (31.6%)	84 (52.8%) 69 (85.2%) 64 (66.7%) 54 (68.4%)	1.49 (1.04 to 2.14) 0.47 (0.25 to 0.87) 1.03 (0.67 to 1.59) Reference	<0.001
**Type of funder** Government, university, international agency Yes No Industry Yes No Foundation, special interest group, or other Yes No	109 (34.0%) 34 (36.6%) 18 (24.0%) 126 (37.1%) 43 (34.7%) 111 (36.9%)	212 (66.0%) 59 (63.4%) 57 (76.0%) 214 (62.9%) 81 (65.3%) 190 (63.1%)	0.95 (0.70 to 1.29) Reference 0.65 (0.42 to 0.99) Reference 0.94 (0.71 to 1.25) Reference	0.7333 0.0315 0.6680
**Impact factor** ^e^	4.6 (2.8, 12.0)	6.0 (3.9, 20.8)	NA	0.0014^f^
**Patient/public engagement** Yes No or unclear	15 (38.5%) 129 (34.3%)	24 (61.5%) 247 (65.7%)	1.12 (0.74 to 1.71) Reference	0.6040
**Other stakeholder engagement** Yes No or unclear	17 (33.3%) 127 (34.9%)	34 (66.6%) 237 (65.1%)	0.96 (0.63 to 1.44) Reference	0.8369
**Publication Year** 2014–2015 2016–2017 2018–2019	30 (28.0%) 68 (41.2%) 46 (32.2%)	77 (72.0%) 97 (58.8%) 97 (67.8%)	Reference 1.47 (1.03 to 2.09) 1.15 (0.78 to 1.69)	0.6390^g^

^a^*n* = 2 trials with unclear use of PROs were included in this group.

^b^Chi-squared test unless otherwise indicated.

^c^Categories obtained from ClinicalTrials.gov and grouped as follows: Clinical (Drug, Device, Biological, Procedure, Radiation, Genetic, Combination Product, Diagnostic Test); Dietary and behavioral (Dietary Supplement, Behavioral); Other (Other).

^d^Categories obtained from ClinicalTrials.gov and grouped as follows: Treatment (Treatment); Health Services Research (Health Services Research); Other (Prevention, Diagnostic, Supportive Care, Screening, Basic Science, Educational/Counseling/Training, Other).

^e^Median (Q1, Q3).

^f^Wilcoxon rank sum test; median, Q1, Q3.

^g^Cochran–Armitage test for trend.

LMIC, low- or middle-income country; PRO, patient-reported outcome.

**Table 4:** Trial characteristics associated with use of PROs as primary or secondary outcomes, compared to not used.

	PROs as primary, coprimary, or secondary outcome (*N =* 235)	PROs not used^a^ (*N =* 180)	Prevalence ratio (95% CI)	*P* value^b^
**Trial age group** ≤18 years >65 years Other or mixture	14 (42.4%) 22 (44.0%) 199 (59.9%)	19 (57.6%) 28 (56.0%) 133 (40.1%)	0.71 (0.47 to 1.06) 0.73 (0.53 to 1.02) Reference	0.0056
**Study setting** Clinical Nonclinical or other	187 (57.0%) 50 (56.2%)	141 (43.0%) 39 (43.8%)	1.01 (0.83 to 1.25) Reference	0.8882
**Trial design** Individually randomized Cluster randomized	171 (64.8%) 66 (43.1%)	93 (35.2%) 87 (56.9%)	1.50 (1.23 to 1.84) Reference	<0.001
**Country of recruitment** LMIC only Non-LMIC or mixture	16 (39.0%) 221 (58.8%)	25 (61.0%) 155 (41.2%)	0.66 (0.45 to 0.98) Reference	0.0153
**Region** North America only Europe only Other or mixture	106 (54.6%) 87 (69.0%) 44 (45.4%)	88 (45.4%) 39 (31.0%) 53 (54.6%)	1.20 (0.94 to 1.55) 1.52 (1.19 to 1.95) Reference	0.0013
**Type of intervention**^c^ Clinical Dietary or behavioral Other or mixture	60 (54.1%) 93 (60.0%) 82 (55.0%)	51 (45.9%) 62 (40.0%) 67 (45.0%)	0.98 (0.78 to 1.23) 1.09 (0.90 to 1.32) Reference	0.5568
**Primary purpose**^d^ Treatment Prevention Health services research Other	109 (69.4%) 27 (34.6%) 50 (54.3%) 51 (56.7%)	48 (30.6%) 51 (65.4%) 42 (45.7%) 39 (43.3%)	1.23 (0.99 to 1.51) 0.61 (0.43 to 0.87) 0.96 (0.74 to 1.24) Reference	<0.001
**Type of funder** Government, university Yes No Industry Yes No Foundation, special interest group, or other Yes No	188 (58.4%) 49 (51.6%) 68 (54.4%) 169 (57.9%) 33 (43.4%) 204 (59.8%)	134 (41.6%) 46 (48.4%) 57 (45.6%) 123 (42.1%) 43 (56.6%) 137 (40.2%)	1.13 (0.91 to 1.40) Reference 0.94 (0.78 to 1.13) Reference 0.73 (0.55 to 0.95) Reference	0.2392 0.5114 0.0090
**Impact factor** ^e^	5.1 (3.1, 15.3)	6.0 (3.9, 20.8)	NA	0.0458^f^
**Patient/public engagement** Yes No or unclear	26 (66.7%) 211 (55.8%)	13 (33.3%) 167 (44.2%)	1.19 (0.94 to 1.52) Reference	0.1929
**Other stakeholder engagement** Yes No or unclear	28 (53.8%) 209 (57.3%)	24 (46.2%) 156 (42.7%)	0.94 (0.72 to 1.23) Reference	0.6419
**Year** 2014–2015 2016–2017 2018–2019	59 (55.1%) 100 (60.6%) 78 (53.8%)	48 (44.9%) 65 (39.4%) 67 (46.2%)	Reference 1.10 (0.89 to 1.36) 0.98 (0.78 to 1.23)	0.7394^g^

^a^*n* = 2 trials with unclear use of PROs were included in this group.

^b^Chi-squared test unless otherwise indicated.

^c^Categories obtained from ClinicalTrials.gov and grouped as follows: Clinical (Drug, Device, Biological, Procedure, Radiation, Genetic, Combination Product, Diagnostic Test); Dietary and behavioral (Dietary Supplement, Behavioral); Other (Other).

^d^Categories obtained from ClinicalTrials.gov and grouped as follows: Treatment (Treatment); Health Services Research (Health Services Research); Other (Prevention, Diagnostic, Supportive Care, Screening, Basic Science, Educational/Counseling/Training, Other).

^e^Median (Q1, Q3).

^f^Wilcoxon rank sum test.

^g^Cochran–Armitage test for trend.

LMIC, low- or middle-income country; PRO, patient-reported outcome.

### Sample size determinations

Details about sample size determinations for all trials as well as those with PROs as primary or coprimary outcomes are presented in Table 5. Among the 144 trials with a PRO as primary or coprimary outcome, 126 (87.5%) reported a sample size calculation, of which 4 based the calculation on an outcome not clearly identified as primary, and 5 had a coprimary PRO but the clinical outcome was used to determine the ultimate sample size. Therefore, 117 (81.3%) trials provided a sample size or power calculation for the primary PRO. Among the 117 trials with a PRO determining the sample size, 71 (60.7%) justified the target difference while 46 (39.3%) did not provide a justification. The most common methods for justifying the target difference were pilot studies (31; 26.5%), standardized effect size (20; 17.1%), or evidence review (16; 13.7%); patient or stakeholder opinion was used to justify the target difference in 8 (6.8%) of these trials. The target difference was classified as an important difference in 18 (15.4%), a realistic difference in 37 (31.6%) and both realistic and important in 12 (10.3%); in 50 (42.7%) the basis for the choice of target difference was unclear. Adjustment for attrition in the sample size calculation was reported for 72 (61.5%) trials.

**Table 5:** Sample size determination and target difference justifications for all trials and those with primary or coprimary PROs.

Characteristic	Frequency (%)	
	Trials with PRO as primary/coprimary	All trials
**Was a sample size or power calculation provided?** Yes, based on primary or coprimary outcome Yes, based on other outcome No	***N* = 144** 122 (84.7%) 4 (2.8%) 18 (12.5%)	***N =* 415** 358 (86.3%) 15 (3.6%) 42 (10.1%)
**When a sample size or power calculation was provided for the primary outcome, was the target difference justified?**^b^ Yes Anchor Distribution Standardized effect size Patient/stakeholder consultation (“opinion seeking”) Review of the evidence base Pilot study Authors specified difference was important No Target difference not justified Calculated power based on available sample size Target difference not specified	***N* = 117** ^a^	***N* = 358**
	71 (60.7%) 6 (5.1%) 5 (4.3%) 20 (17.1%) 8 (6.8%) 16 (13.7%) 31 (26.5%) 1 (0.9%) 46 (39.3%) 39 (33.3%) 4 (3.4%) 3 (2.6%)	187 (52.2%) 6 (1.7%) 5 (1.4%) 25 (7.0%) 18 (5.0%) 49 (13.7%) 100 (27.9%) 7 (2.0%) 171 (47.8%) 151 (42.2%) 15 (4.2%) 5 (1.4%)
When a sample size or power calculation was provided for the primary outcome, what type of target difference was used? Important Realistic Both important and realistic Not specified or unclear	*N* = 117^a^	*N* = 358
	18 (15.4%) 37 (31.6%) 12 (10.3%) 50 (42.7%)	39 (10.9%) 132 (36.9%) 20 (5.6%) 167 (46.6%)
When a sample size or power calculation was provided for the primary outcome, was it adjusted for attrition? Yes No Other or unclear	*N* = 117^a^	*N* = 358
	72 (61.5%) 44 (37.6%) 1 (0.9%)	157 (43.9%) 197 (55.0%) 4 (1.1%)

^a^Trials with coprimary clinical and PROs but where the clinical outcome was used to determine the sample size (*n =* 5) and trials presenting sample size calculation on an outcome not clearly identified as primary (*n* = 4) were excluded.

^b^More than one method may have been used within one study.

PRO, patient-reported outcome.

## Discussion

### Summary of key findings

Among a large sample of pragmatic RCTs published 2014 to 2019, the use of PROs and prevalence of reporting on patient or public engagement were low. Just over a third chose PROs as primary or coprimary outcomes, while just over half chose PROs as either primary or secondary outcomes. Virtually, no trial cited patient or other stakeholder consultation in the choice of the primary outcome, even when the outcome was a PRO. Pediatric trials and trials in older adults, as well as trials conducted in LMICs had comparatively lower prevalence of use of PROs. Target differences for primary PROs were often not justified in sample size calculations; when a justification was provided, it was rarely based on patient or stakeholder consultation.

### Comparison with other studies

To our knowledge, no other study has described use of PROs and sample size reporting in a general sample of pragmatic trials. However, several studies have identified similar patterns of inadequate use of best practices, infrequent patient engagement [26], and poor reporting among trials including PROs. A scoping review of 44 trials with patient-important or patient-relevant outcomes identified that only 36% of studies included patients or stakeholders in determining outcomes [27]. Another review of 75 protocols for trials including PROs showed that PRO-specific sample size justifications were provided only 51% of the time, and 61% of PRO-specific items from the SPIRIT-PRO guidelines were incomplete [8]. Several reviews of cancer trials, which included PROs as outcomes [28–31], revealed that adherence to guidelines from SPIRIT [7] and ISOQOL [5] for reporting on PROs in clinical trials was consistently suboptimal; authors often did not adequately describe justifications for choice of PRO or sample size calculations. Collectively, these studies corroborate many of our findings across settings and clinical areas. Although we did not identify a significant increase in use of PROs over time, others have identified an increase in PROs used in trials registered at ClinicalTrials.gov between 2004 and 2013 [15,16]. An increase was also observed in the Australia and New Zealand Clinical Trials Registry between 2005 and 2017, with 64% of trials registered in 2016 including at least one PRO [32]. It is possible that our observation window between 2014 and 2019 was too narrow to detect a trend or that the use of PROs has plateaued in recent years. Notably, the CONSORT-PRO extension for trial reports was published in 2013 [8], while the SPIRIT-PRO extension for trial protocols was published later, in 2018 [7]. One might expect a gradual increase in PRO reporting from 2013 onwards, but this was not observed in our data.

### Strengths and limitations

There are some key strengths of this study. Identification of pragmatic trials in the literature is challenging. Other reviews of pragmatic trials have used limited or arbitrary search terms to identify pragmatic trials [33,34]. We used a published search filter [18], which relied on specific terms and phrases in the title and abstract shown to be associated with pragmatism. We used this approach because there are no reporting guidelines requiring authors to label their trials as pragmatic in the title or abstract and we wanted to include a broader range of trials with pragmatic intention. Our search resulted in a large sample of trials across a wide range of clinical areas, study settings, and patient populations, which allowed us to examine characteristics associated with use of PROs more broadly.

Our study had limitations. From our larger database of trials, we selected the subset that were registered in ClinicalTrials.gov. Although ClinicalTrials.gov registration has become widespread and even federally mandated in the United States [35], our sample may have captured more American studies than those conducted elsewhere. We focused on information provided in the primary trial reports and did not retrieve information provided in trial protocols, which means that misclassification was possible. Our analyses of factors associated with use of PROs were exploratory and considered characteristics individually; we did not conduct multivariable analyses to identify factors independently associated with PROs. Finally, many of our trial characteristics were downloaded directly from CT.gov, thus any inaccurate classifications by trial authors in CT.gov may have influenced our findings.

### Implications for research and practice

Given that pragmatic trials are intended to inform clinical practice and incorporate patient perspectives [2], the prevalence of use of PROs and patient and public engagement, especially among trials with PROs, was surprisingly low. Our search covered the period 2014 to 2019, which is several years after the establishment of various patient engagement strategies in the UK [36], Canada [37], and USA [38], and publication of the CONSORT-PRO guidelines in 2013 [8]. One explanation for lower than expected prevalence of PROs could be related to the use of routinely collected data often associated with pragmatic trials; availability of PROs in such databases remains rare [39]. However, a defining characteristic of pragmatic trials is that the key results should be useful to decision-makers. Choosing the right outcome is therefore even more important than outcome source. Where an appropriate outcome to inform decision-making is not available routinely, pragmatic trials need to collect data directly from participants but in a way that does not interfere too much with routine clinical practice [2]. A possible explanation for the low prevalence of patient and public engagement is poor quality of reporting; authors are encouraged to report patient engagement, especially concerning PROs [40], using tools such as the Guidance for Reporting the Involvement of Patients and the Public (GRIPP2) checklist [41].

Not surprisingly, given the low prevalence of patient or public engagement, involvement of patients and stakeholders in determining target differences for sample size calculations was rare. The minimal important difference has been defined as “the smallest difference in score in the outcome of interest that informed patients perceive as important, either beneficial or harmful, and which would lead the patient or clinician to consider a change in the management” [42,43]. Best practices given by ISOQOL [44] recommend establishing target differences for PROs to enhance the applicability of their use in clinical trials to care settings [10]. However, patient and public engagement in determining target differences is not included in these or other guidelines [22], though it would seem prudent for greater emphasis to be placed on this type of engagement [45]. Although obtaining input from patients about numerical aspects of trials such as target differences may raise particular challenges (especially in the case of more complex analyses and types of effect sizes), patients and members of the public have expressed interest in being included in discussing and contributing to the definition of the target difference used, which they believed would improve the transparency of research [46]. Patients, researchers, and statisticians have acknowledged that including patients in the actual statistical analysis may not be an efficient use of resources, but engagement while developing the assumptions required for determining the target difference, for example, could be one way to ensure that the patient perspective is captured [46,47]. Yet, there are gaps in the literature with important conceptualization to be done in this space. Guidance for determining meaningful differences in PROs across patient groups, clinical diagnoses, and treatment contexts is available, but there is uncertainty about how to best involve patients in its application [48,49]. For example, a treatment with less functional impairment might be desirable if the clinical effectiveness is maintained, but no longer acceptable if the clinical effectiveness is reduced. Though it makes sense in some study contexts to include clinical measures as primary outcomes with PROs as secondary outcomes, statistical power to detect a meaningful difference in secondary outcomes is often insufficient or not considered. Researchers might benefit from guidance for designing trials when clinical and patient reported outcomes are selected as coprimary outcomes, including how to resolve differences in sample size requirements, how to deal with multiplicity, and how to engage with patients to inform and justify the choice of target difference.

We recommend that funding agencies, institutions, and journal editors adopt explicit policies to encourage researchers to consider incorporating PROs and engage patients in pragmatic trials. Journal editors and peer reviewers should require explicit reporting of whether and how patients and members of the public were engaged in the trial design and conduct and require that authors provide clearer justification for the choice of primary outcome and the target differences in reporting results from pragmatic trials. Journals could mandate that authors use SPIRIT-PRO checklist or protocol template [40] or report patient and public engagement [50] to improve reporting and transparency around use of PROs. Trialists and methodologists should give greater consideration to choosing coprimary outcomes, with adjustment for multiplicity as appropriate, in trials where both clinical and patient perspectives are important in informing treatment decisions. Institutions can provide resources to promote and support the identification and recruitment of patient partners in their research, methodological and analytical support for use of PROs in pragmatic trials, and support for the development of tools to help patient partners participate fully in research. Researchers can equip patient partners with tools to understand and contribute to the design of studies that include PROs, such as the web tool created by Cruz Rivera and colleagues [51], which aims to supports dissemination and uptake of the SPIRIT-PRO extension by patient partners. Given that researchers can be incentivized to use specific methods or measure certain outcomes according to funding or publication calls, there is an opportunity for funders to support and promote the use of patient and public engagement and PROs in pragmatic trials. These strategies are likely to increase the applicability of pragmatic trials to clinical decision-making and patient preferences by encouraging the inclusion of patients and stakeholders in health research.

## Conclusions

In conclusion, PROs were infrequently used as primary outcomes in pragmatic trials published between 2014 and 2019. Patient and stakeholder engagement was rarely reported and was not commonly used when determining the target difference among trials in this review. As pragmatic trials are intended to answer clinically relevant and patient-important questions, they stand to benefit from prioritizing inclusion of patient reported outcomes and patient and stakeholder engagement in the future.

## Supporting information

S1 PRISMA Checklist PRISMA checklist. (DOCX)

S1 Search Filter Electronic search filter to identify pragmatic trials in MEDLINE. (DOCX)

S1 Screening Inclusion and exclusion criteria. (DOCX)

S1 Protocol Study protocol. (DOCX)

S1 Data Extraction Form Data extraction form. (DOCX)

S1 Data Dataset. (CSV)

## Abbreviations

ISOQOL: International Society for Quality of Life Research
JCR: Journal Citation Reports
LMIC: low- or middle-income country
PRO: patient-reported outcome
PROMIS: Patient Reported Outcome Measurement Information System
QOL or HRQOL: quality of life or health-related quality of life
RCT: randomized controlled trial
SJR: SCImago Journal and Country Rank
