# Supplementary material for: Patient-reported outcomes and target effect sizes in pragmatic randomized trials in ClinicalTrials.gov: A cross-sectional analysis
Source: PLoS Med. 2022 Feb 8;19(2):e1003896. doi: 10.1371/journal.pmed.1003896 (PMC8824332; doi:10.1371/journal.pmed.1003896)
Supplement: S1 Search Filter — (DOCX) [file pmed.1003896.s002.docx]

**S1: Electronic search filter to identify pragmatic trials in MEDLINE^[[1]](#endnote-1)^**

| **#** | **Search Statement** |
| --- | --- |
|  | **Trial design terms** |
| 1 | (((pragmatic$ OR naturalistic OR real world OR real life OR unblinded OR unmasked OR cluster OR step$ wedge$ OR point of care OR factorial OR switchback OR switch back OR phase 4 OR phase IV) adj10 (study OR trial)) OR (practical trial OR effectiveness trial OR ((cluster$ or communit$) adj2 randomi$))).tw. |
|  | **Trial attribute terms** |
| 2 | (general practice$ OR primary care OR registry based OR health record$ OR medical record$ OR EHR OR EMR OR administrative data OR routinely collected data OR (communit$ adj2 intervention$) OR quality improvement OR implementation OR decision support OR health service$ OR health system$ OR comparative effectiveness OR CER OR usual care OR evidence based OR practice guideline$ OR (guideline$ adj1 recommend$) OR knowledge translation OR health technology assessment OR HTA OR cost effectiveness OR process evaluation OR economic evaluation OR patient oriented).tw. |
|  | **Limit to records likely to be RCTs** |
| 3 | randomized controlled trial.pt. OR ((comparative effectiveness OR randomi?ed) adj10 (trial OR study)).ti. |
| 4 | (comment on OR phase 1 OR phase I OR phase 2 OR phase II OR non-randomi?ed OR quasi-randomi?ed OR pseudo-randomi?ed).ti. OR (clinical trial, phase I OR clinical trial, phase II OR systematic review OR meta-analysis OR review OR editorial).pt. |
|  | **Include records tagged as pragmatic trials** |
| 5 | pragmatic clinical trial.pt. |
|  | **Sensitivity-maximizing search (combines trial design terms or attribute terms with RCT terms)** |
| 6 | ((1 OR 2) AND (3 NOT 4)) OR 5 |
| 7 | exp Animals/ NOT Humans/ |
| 8 | 6 NOT 7 |

**RCT=randomized controlled trial**

1. Taljaard M, McDonald S, Nicholls SG, Carroll K, Hey SP, Grimshaw JM, Fergusson DA, Zwarenstein M, McKenzie JE. A search filter to identify pragmatic trials in MEDLINE was highly specific but lacked sensitivity. J Clin Epidemiol. 2020 Aug;124:75-84. doi: 10.1016/j.jclinepi.2020.05.003. Epub 2020 May 11. PMID: 32407765. [↑](#endnote-ref-1)
