# Supplementary material for: Patient-reported outcomes and target effect sizes in pragmatic randomized trials in ClinicalTrials.gov: A cross-sectional analysis
Source: PLoS Med. 2022 Feb 8;19(2):e1003896. doi: 10.1371/journal.pmed.1003896 (PMC8824332; doi:10.1371/journal.pmed.1003896)
Supplement: S1 Screening — (DOCX) [file pmed.1003896.s003.docx]

**S1 Screening: Inclusion and exclusion criteria for creation of the large database of pragmatic trials^[[1]](#endnote-1)^**

| **INCLUSION CRITERIA** | | **SPECIFICATION** |
| --- | --- | --- |
| **Health RCT** | A health RCT evaluates interventions aimed at changing subjective or objective measures of individual or group health status, or of processes which lead to changes in health status. Health status is defined as a state of human wellbeing, in individuals or groups, with physical or mental health correlates. | Trial must use randomization (as opposed to quasi-randomization) and must be comparative (i.e., at least one intervention and one control arm, or at least two intervention arms for “comparative effectiveness research”). |
| **Health care RCT** | A health care RCT is one aimed at evaluating changes in the delivery of services to changing health status, or to change processes in the delivery of care that are known to lead to changes in health status. This includes RCTs of treatment, prevention, health promotion, health knowledge or behaviour as well as studies of the implementation, acceptability, efficiency, equity or cost of interventions for treatment, prevention, promotion, health knowledge or behaviour change.  These studies may target individual patients, groups of patients, communities or populations, and/or carers from family, community and health care systems. | Trial must have a target enrolment of at least 100 individuals |
| **EXCUSION CRITERIA** | | **SPECIFICATION** |
| 1 | Not a randomized controlled trial | Trial must use randomization (as opposed to quasi-randomization) and must be comparative (i.e., at least one intervention and one control arm, or at least two intervention arms for “comparative effectiveness research”). |
| 2 | RCT but with <100 target enrolment | Trial must have a target enrolment of at least 100 individuals |
| 3 | Study protocol or design paper | Study does not report trial outcomes or reports only baseline data. Includes study protocols and published statistical analysis plans. |
| 4 | Methods paper – not a trial report | Exclude if the article doesn’t present any results from a trial, e.g., it may only describe the design of a trial without presenting results or it may talk about recruitment difficulties or intervention development only or only a statistical analysis plan. Some studies may be a trial themselves e.g. the trial is about the best consent approach within a trial and they test two consent methods. |
| 5 | Not health research | Excluded trials include those of purely educational intervention in school-based settings that don’t have a distinct health link. For example, a trial testing two different Mathematics curricula or trials assessing interventions to reduce bullying, but there is no direct health link (e.g., does not involve school nurses and does not assess health outcomes, e.g., anxiety.) There must be a clear link to health research. Other exclusion examples include trials testing new ways of sweeping the floor, or new administrative approaches of contacting patients, or recruiting patients into trials, or improving data quality, or new hiring practices. Unless there was a clear and direct route by which this would impact on patient health outcomes, the trial is not eligible (lack of direct relevance to patient health). |
| 6 | Pilot or feasibility study | If the trial is obviously labelled as a pilot or feasibility study, it is ineligible. If the conclusions simply refer to “feasibility”, but also gives results for a primary health or health care outcome and it meets all the other criteria for inclusion the trial would be included. |
| 7 | Not a pragmatic intent | Trial that is obviously not of pragmatic orientation. For example, trials that focused on isolating a biological impact of an intervention without a clear clinical implication, or that did not assess clinical outcomes, were deemed more likely to not to have a pragmatic orientation. |
| 8 | Non-primary trial report | Any indication in the manuscript that it is not the primary trial report (meaning, the analysis of the primary trial outcome), indicates exclusion. Examples of non-primary reports include the analysis of an outcome clearly identified as a secondary trial outcome; subgroup analysis with primary trial results reported elsewhere; long-term follow-up (with primary endpoint reported previously), process analysis, mediation analysis, sensitivity analysis (if reported separately to primary outcome).** |
| 9 | Educational intervention of health professionals with no real patients or patient data | Studies randomizing clinicians to different vignettes and then surveying them to assess their response are excluded as they do not involve real patients. Other trials excluded here are: trials involving only manikins or simulations (e.g., for training providers to do CPR) or trials of purely educational (provider) interventions which do not measure patient data. For example, if a trial tests two different Medical curricula or outcomes are exclusively measures of clinical knowledge measured in clinicians or medical residents, the trial is not eligible. |
| 10 | Other (specify) |  |

** Trial registration information was used to facilitate identification of the primary trial report from among multiple publications from the same trial. Studies were also compared by first and senior authors and studies that reported the same trial were flagged. Articles flagged as potential non-primary reports were also scrutinized for any explicit statement referring to “primary results being published previously” or that the present article was presenting a secondary analysis. When multiple publications were associated with the same clinical trial registration, a decision had to be made about whether any could be considered the primary trial publication. In cases of uncertainty, the primary outcome in the registry was used to guide the decision. In the case of complex study designs, such as factorial designs with interventions reported separately, the article reporting on the first listed intervention or outcome reported in the registry was selected as the primary report. Finally, our search identified several Health Technology Assessment (HTA) reports, unique to studies funded by the UK National Institutes of Health Research. When the HTA report was the only report of the trial retrieved it was retained as the primary publication; otherwise, the associated journal publication of the trial was retained as the primary publication.

1. Nicholls SG, Carroll K, Hey SP, Zwarenstein M, Zhang JZ, Nix HP, Brehaut JC, McKenzie JE, McDonald S, Weijer C, Fergusson DA, Taljaard M. A review of pragmatic trials found a high degree of diversity in design and scope, deficiencies in reporting and trial registry data, and poor indexing. J Clin Epidemiol. 2021 Sep;137:45-57. doi: 10.1016/j.jclinepi.2021.03.021. Epub 2021 Mar 28. PMID: 33789151. [↑](#endnote-ref-1)
