# Supplementary material for: Patient-reported outcomes and target effect sizes in pragmatic randomized trials in ClinicalTrials.gov: A cross-sectional analysis
Source: PLoS Med. 2022 Feb 8;19(2):e1003896. doi: 10.1371/journal.pmed.1003896 (PMC8824332; doi:10.1371/journal.pmed.1003896)
Supplement: S1 Protocol — (DOCX) [file pmed.1003896.s004.docx]

**S1: Study Protocol**

**Overall objective:** To describe the prevalence and types of patient-reported outcomes (PROs) in pragmatic clinical trials registered in ClinicalTrials.gov and identify factors associated with using PROs in such trials.

**Specific objectives:**

1. What is the prevalence of using PROs in pragmatic trials?
2. What is the prevalence of using PROs as *primary/co-primary and secondary* outcomes in pragmatic trials?
3. What types of PROs are used in pragmatic clinical trials according to PROMIS categories?^[[1]](#footnote-1)^
4. Does the use of PROs as primary/co-primary or secondary outcomes vary across:
   1. Study settings? (e.g., primary care, hospital care, community settings)
   2. Trial design? (e.g., individual versus cluster randomized)
   3. Country? (e.g., LMIC vs. non-LMIC, North America, Europe, etc)
   4. Types of interventions? (according to CT.gov categories)
   5. Primary purposes of interventions? (according to CT.gov categories)
   6. Clinical/disease areas? (according to MeSH information)
   7. Type of funder?
   8. Journal impact factor? General, specialty, medical/surgical, other?
5. Is the use of PROs associated with patient or stakeholder engagement?
6. Has there been an increase in the use of PROs as primary outcomes over the past 5 years?
7. How are target differences determined for PROs, when used as primary outcomes, and how are sample sizes justified?
8. How common is the use of PROs and clinical outcomes as co-primaries in pragmatic trials, and in those situations how is the ultimate sample size determined?
9. How do studies justify selection of PROs, and provide evidence of patient relevance?

**Definitions:** PRO is defined as any report of the subjective status of a patient’s health condition or response to an intervention that comes directly from the patient or their proxy, without interpretation of the patient’s response by a clinician or anyone else, for example, health-related quality of life, symptoms, severity, utilities, pain, satisfaction. A PRO is in contrast to a Clinician-reported Outcome, Observer-reported Outcome (e.g., parent/spouse/caregiver), or Performance Outcome

**Data source:** We will use the subset of our large pragmatic trials database, i.e., N=415 self-declared pragmatic trials that are registered in CT.gov. The reason we want to analyze this subset is because we already have descriptive information downloaded from CT.gov and some relevant items have already been extracted for the main project.

**Methods:** An electronic search filter in MEDLINE was used to identify primary reports of RCTs in health research with a pragmatic intent, published 2014-2019. A subset with a corresponding registration in ClinicalTrials.gov (CT.gov) and explicitly identified as “pragmatic” anywhere in the title, abstract or main text was identified. Trial descriptors were downloaded from CT.gov. We will extract additional descriptors, information about PROs, and sample size calculations from each report.

**Analysis:** Data will be summarised descriptively using count and percentages. Variation in use of PROs across trial characteristics will be examined using simple tests of association.

1. Cella D, Riley W, Stone A, et al. The Patient-Reported Outcomes Measurement Information System (PROMIS) developed and tested its first wave of adult self-reported health outcome item banks: 2005-2008. *Journal of clinical epidemiology.* 2010;63(11):1179-1194 [↑](#footnote-ref-1)
