# Supplementary material for: Patient-reported outcomes and target effect sizes in pragmatic randomized trials in ClinicalTrials.gov: A cross-sectional analysis
Source: PLoS Med. 2022 Feb 8;19(2):e1003896. doi: 10.1371/journal.pmed.1003896 (PMC8824332; doi:10.1371/journal.pmed.1003896)
Supplement: S1 Data Extraction Form — (DOCX) [file pmed.1003896.s005.docx]

**S1: Data Extraction Form**

1. Extractor name:
2. Total number of eligible participants enrolled (trial size at the start of the trial (NOTE: Look in abstract or flow diagram and indicate the number of **eligible** participants randomised. In most RCTs, eligibility status is determined and consent obtained before randomisation in which case we simply extract the number randomised. For a select number of trials, it may not be possible to establish eligibility before randomisation in which case we extract the number after eliminating those that were later determined to be ineligible or who did not consent.
   For CRTs we want the total number of eligible individuals contributing to the trial at the lowest level of the hierarchy, i.e., patients or citizens. If the CRT only reports aggregate data, e.g., “event rates” we want the population size contributing to the denominator – not the numerator. This is often the “offset term” in a Poisson regression.
   In CRTs with multiple phases (e.g., cross-over, stepped wedge, pre and post design), we want the total number of unique participants contributing to the trial in all phases.
   Leave the item missing if trial size is unknown or not reported but add an explanation in the comment box).
3. Type of setting in which intervention was delivered:

| ^1^ | Clinical: Primary care (e.g., primary care clinics, primary care providers) | |
| --- | --- | --- |
| ^2^ | Clinical: Hospital or specialist care (e.g., hospitals, hospital wards, specialist providers) | |
| ^3^ | Clinical: Nursing homes, long-term care | |
| ^4^ | Public health: Community or residential setting | |
| ^5^ | Public health: Schools | |
| ^6^ | Public health: Workplaces | |
| ^7^ | Other (doesn’t fit any existing categories, multiple, or any additional details |  |

1. Unit of randomisation:

| ^1^ | Individually randomised |
| --- | --- |
| ^2^ | Cluster randomised |

1. Country of study recruitment (for identification of development status, use <https://data.worldbank.org/country>) (Select all that apply):
2. Canada
3. USA
4. UK
5. Other European Union (EU) country
6. Australia or New Zealand
7. Low or Middle Income Country (LMIC)
8. Other developed
9. Did the authors clearly identify a primary outcome in the trial report? (NOTE: Trial report should have one or a limited number of clearly identified outcomes as “primary”).

| ^1^ | Yes: one primary outcome clearly identified |
| --- | --- |
| ^2^ | Yes: two or three co-primary outcomes clearly identified |
| ^3^ | No: none or unclear (e.g., simply lists many outcomes without identifying the primary outcome) |

7.1. Any comments or explanation about Q7? (Add any clarification about the primary outcome data extraction here)

1. What was/were the trial primary outcomes in the trial report? (Note: A single primary outcome or two or three co-primary outcomes must be identified for each trial to facilitate extraction. Use the following hierarchy: First primary efficacy outcome stated by authors; if no primary outcomes specified, use outcome in sample size calculation; if sample size calculation not reported or reported for a sub-study only, use first outcome listed under ‘Objectives’)

|  |
| --- |

1. Does the trial include one or more patient-reported outcomes (PROs) as primary or secondary outcomes? Note: this should be an **outcome** of the trial – not a process measure, measure of protocol adherence, or covariate. (PRO: “Any report of the subjective status of a patient’s health condition or response to an intervention that comes directly from the patient or their proxy, without interpretation of the patient’s response by a clinician or anyone else, for example, health-related quality of life, symptoms, severity, utilities, pain, satisfaction. A PRO is in contrast to a Clinician-reported Outcome, Observer-reported Outcome (e.g., parent/spouse/caregiver), or Performance Outcome – see FDA definitions document. An example of a clinician-reported outcome is a standardised instrument administered by clinician, with interpretation by the clinician, and routinely documented in chart. Example: NRS pain is a PRO as it is subjective and not subject to interpretation by the clinician.)

| \| ^1^ \| Yes – PRO is a primary or co-primary outcome \| \| \| --- \| --- \| --- \| \| ^2^ \| Yes – PRO is a secondary outcome only (not primary) \| \| \| ^3^ \| No – There are no PROs \| \| \| ^4^ \| Unclear or other (specify) (e.g., PROs assessed on a subset of patients only, or unclear whether PRO is primary or secondary) \|  \| |
| --- | --- | --- | --- | --- | --- | --- | --- | --- | --- | --- | --- | --- |

1. If the trial has multiple co-primary outcomes (i.e., Q7=2), are they a mix of PROs and non-PRO outcomes?

| \| ^1^ \| Yes \| \| --- \| --- \| \| ^2^ \| No \| \| ^3^ \| NA – trial does not have multiple co-primary outcomes \| |
| --- | --- | --- | --- | --- | --- | --- |

10.1. Any explanation or comment about Q10?

1. For any trial with at least one PRO as primary or co-primary (i.e., Q9= 1), what type of PRO is used according to PROMIS categories? (Note: Select all that apply; trials with two or more co-primary PROs can have multiple types). If a trial simply lists main outcomes without clearly identifying one or two as “PRIMARY”, we can still complete this item

| ^1^ | HRQoL or QoL | |
| --- | --- | --- |
| ^2^ | Functional status | |
| ^3^ | Symptoms and symptom burden (including pain) | |
| ^4^ | Health behaviours (including substance use) | |
| ^5^ | Patient experience (evaluations of patient satisfaction, patient motivation and activation, and patient reports of their actual experiences) | |
| ^6^ | Other (specify) |  |
| ^7^ | NA – trial does not have PRO as primary outcome | |

1. For any trial with at least one PRO as secondary (i.e., Q9= 2), what type of PRO is used according to PROMIS categories? (Note: Select all that apply; trials with two or more co-primary PROs can have multiple types). If a trial simply lists main outcomes without clearly identifying one or two as “PRIMARY”, we can still complete this item

| ^1^ | HRQoL or QoL | |
| --- | --- | --- |
| ^2^ | Functional status | |
| ^3^ | Symptoms and symptom burden (including pain) | |
| ^4^ | Health behaviours (including substance use) | |
| ^5^ | Patient experience (evaluations of patient satisfaction, patient motivation and activation, and patient reports of their actual experiences) | |
| ^6^ | Other (specify) |  |
| ^7^ | NA – trial does not have PRO as secondary outcome | |

1. As per CONSORT extension for PRO: If PRO is primary or co-primary, is there any justification provided for including a PRO as an outcome?

| \| ^1^ \| Yes – reference to other literature or explanation provided (e.g. “Patient activation “emphasises patients’ willingness and ability to take independent actions” [30] by understanding their “role in the care process and having the knowledge, skill, and confidence to manage one’s health and health care [31]”) \| \| \| --- \| --- \| --- \| \| ^2^ \| Yes – patient consultation (e.g. “During meetings with patient and caregiver advisers, knowledge about risk of acute coronary syndrome emerged as the outcome of greatest importance, so we selected patient knowledge as the primary outcome”) \| \| \| ^3^ \| Yes – other stakeholder consultation \| \| \| ^4^ \| No \| \| \| ^5^ \| Unclear or other (specify) \|  \| \| ^6^ \| NA – trial does not have PROs as primary outcome(s) \| \| |
| --- | --- | --- | --- | --- | --- | --- | --- | --- | --- | --- | --- | --- | --- | --- | --- | --- | --- | --- |

1. Does the trial explicitly report on patient or public engagement or elicitation of patient or public perspectives in the research? Note: Patient engagement is defined as “meaningful and active collaboration in governance, priority setting, conducting research and knowledge translation”. Hint: search for “engagement”, “consultation”, “advisory”, “perspective”, “stakeholder”, “committee” or “interview”.

| ^1^ | Yes | |
| --- | --- | --- |
| ^2^ | No | |
| ^3^ | Unclear or other (please explain) |  |

1. Does the trial explicitly report on any other stakeholder engagement or elicitation of stakeholder perspectives on the research? Note: This could include knowledge user, decision-maker or policy perspectives. It should be explicitly described. Hint: search for words like “engagement”, “consultation”, “advisory”, “perspective”, “stakeholder”, “committee” or “interview”.

| ^1^ | Yes | |
| --- | --- | --- |
| ^2^ | No | |
| ^3^ | Unclear or other (please explain) |  |

1. Does the trial report include a sample size calculation/justification or any statement about power?

| ^1^ | Yes, for the identified (co)primary trial outcome (or one of the co-primaries) in Q7 | |
| --- | --- | --- |
| ^2^ | Yes, but not for the (co)primary trial outcome (e.g., another outcome or primary outcome unknown) | |
| ^3^ | No | |
| ^4^ | Other (explain) |  |

1. If Q16=Yes, what was the target sample size in the sample size calculation section of the trial report (number of individuals)? Please be sure to indicate the number of participants at the individual-level – not the number of clusters.
2. If trial includes a sample size calculation (i.e., Q16=1 or 2), what method was used to determine the target difference according to categories by Cook et al.^[[1]](#footnote-1)^? (Note: If the trial has co-primaries, choose the outcome that ultimately determined the sample size)

| ^1^ | Anchor (i.e., the outcome of interest is “anchored” by using either a patient’s or health professional’s judgement to define an important difference. This may be achieved by comparing before and after treatment and then linking this change to participants who had an improvement/deterioration. Alternatively, a contrast between patients can be made to determine a meaningful difference) | | |
| --- | --- | --- | --- |
| ^2^ | - Distribution (i.e., a value that is larger than the inherent imprecision in the measurement and therefore likely to represent a minimal level for a meaningful difference) | | |
| ^3^ | - Health economic (i.e., typically involves defining a threshold value for the cost of a unit of health effect that a decision-maker is willing to pay and using data on the differences in costs, effects and harms to make an estimate of relative efficiency. This can be based upon a net benefit or value of information approach which seeks to take into account all relevant aspects of the decision and can be viewed as implicitly determining a target difference) | | |
| ^4^ | - Standardised effect size (i.e., the magnitude of the effect upon a standardised scale is used to define the value of the difference. For a continuous outcome, the standardised difference (Cohen’s d; eg: 0.2, 0.5, 0.8) can be used. Binary or survival (time-to-event) outcome metrics (e.g. an odds, risk or hazard ratio) can be used, though no widely recognised cut-offs exist. Cohen’s cutoffs approximate to odds ratios of 1.44, 2.48 and 4.27 respectively) | | |
| ^5^ | Stakeholder/patient opinion (i.e., formal approaches for determining the target difference on the basis of eliciting (often a health professional’s although it can be patient’s or other’s) opinion. Possible approaches include forming a panel of experts, surveying the membership of a professional or patient body or interviewing individuals) | | |
| ^6^ | Evidence review (target difference derived using current evidence on the research question. Ideally, this would be based upon a systematic review of RCTs, and possibly meta-analysis, of the outcome of interest which directly addresses the research question at hand. In the absence of randomised evidence, evidence from observational studies could be used in a similar manner. An alternative approach is to undertake a review of studies in which an important difference was determined) | | |
| ^7^ | Pilot study or realistic difference (A pilot (or preliminary) study may be carried out where there is little evidence, or even experience, to guide expectations and determine an appropriate target difference for the trial. The planned definite study can be carried out in miniature in order to inform the design of the future study. In a similar manner, a Phase II study could be used to inform a Phase III study) | | |
| ^8^ | Authors specified difference was important | | |
| ^9^ | No target difference stated | |  |
| ^10^ | Calculated the detectable difference given the sample size | |  |
| ^11^ | Target difference not justified | |  |
| ^12^ | Unclear or other (specify) |  |  |

18.1 Was the target difference driven by an important or realistic difference?

| ^1^ | Realistic | |
| --- | --- | --- |
| ^2^ | Important | |
| ^3^ | Both |  |
| ^4.^ | Unclear or NA | |

18.2 For trials with PROs as primary or co-primary outcomes (i.e., Q9= 1), what was the target difference specified, if applicable?

|  |
| --- |

1. If the trial has multiple co-primary outcomes (i.e., if Q7=2), includes a sample size calculation (i.e., Q16=1), and is a mixture of PRO and non-PRO co-primary outcomes (i.e., Q10=Yes), how was sample size determined?

| ^1^ | PRO drove the ultimate sample size | |
| --- | --- | --- |
| ^2^ | Clinical outcome drove the ultimate sample size | |
| ^3^ | Unclear or other (specify) |  |
| ^4.^ | NA | |

1. Any general comments about Q18, e.g., were sample sizes presented for both types of outcomes, were there differences between the sample sizes and how was this resolved (e.g., calculated sample size for both outcomes and chose the most conservative)?
2. Does the sample size calculation include an adjustment for attrition?

| ^1^ | Yes | |
| --- | --- | --- |
| ^2^ | No | |
| ^3^ | Unclear or other (explain) |  |
| ^4.^ | NA – no sample size calculation | |

1. Primary purpose (Note: this field will be copied from the existing field ct_primary_purpose. It is complete for the vast majority of trials, but if it is missing, please add your classification or add an explanation under “other” if you cannot classify it)

| ^1^ | Treatment (treating a disease, syndrome, or condition) | |
| --- | --- | --- |
| ^2^ | Prevention (preventing the development of a disease or health condition) | |
| ^3^ | Diagnostic (identifying a disease or health condition) | |
| ^4^ | Supportive care (maximizing comfort, minimizing side effects, mitigating against a decline in health or function) | |
| ^5^ | Screening (identifying a condition, or risk factors for a condition) | |
| ^6^ | Health Services Research (evaluation the delivery, processes, management, organisation, or financing of healthcare) | |
| ^7^ | Other (specify) (Please add a few words describing “other”) |  |

1. Type(s) of **experimental** interventions (Select all that apply) (NOTE: The information is already extracted but is hidden within the field ct_intervention; please select the relevant categories below and if “other”, add some explanatory text)

| ^1^ | Drug | |
| --- | --- | --- |
| ^2^ | Device | |
| ^3^ | Biological/Vaccine | |
| ^4^ | Procedure /Surgery | |
| ^5^ | Radiation | |
| ^6^ | Genetic (gene transfer, stem cell and recombinant DNA) | |
| ^7^ | Dietary Supplement (e.g., vitamins, minerals) | |
| ^8^ | Combination product (e.g., combining drug and device) | |
| ^8^ | Diagnostic Test (e.g., imaging) | |
| ^9^ | Patient educational or behavioral (e.g., psychotherapy, lifestyle counseling) (NOTE: CT.gov only has behavioural – in that case, check to see whether it is patient behavioural or provider behavioural) | |
| ^10^ | Provider educational or behavioural (Note: New category not in CT.gov) | |
| ^11^ | Other (specify) (Please add specific details for “other”) |  |

1. Who funded the study? *(select all that apply)* (Note: This may be located in the acknowledgements or in a specific funding section.)

| ^1^ | Industry | |
| --- | --- | --- |
| ^2^ | Government agency, international development agency, university, institutes | |
| ^3^ | Foundation, special interest group (e.g., Bill & Melinda Gates Foundation, Alzheimer’s Society, charitable/non-profit organisation) | |
| ^4^ | Other (specify) |  |

1. Was the study a superiority, non-inferiority, or equivalence trial?

| ^1^ | Superiority |
| --- | --- |
| ^2^ | Non-inferiority |
| ^3^ | Equivalence |

1. Did the statistical analysis have a frequentist or Bayesian approach?

| ^1^ | Frequentist |
| --- | --- |
| ^2^ | Bayesian |

1. Cook, J.A., Hislop, J., Altman, D.G. *et al.* Specifying the target difference in the primary outcome for a randomised controlled trial: guidance for researchers. *Trials* **16,**12 (2015). https://doi.org/10.1186/s13063-014-0526-8 [↑](#footnote-ref-1)
